# Physical exercise and adolescent negative emotions: indirect associations via stress perception and emotional sensitivity

**DOI:** 10.1186/s40359-026-04093-5

**Published:** 2026-02-09

**Authors:** Yang Xin, Lu Liuheng

**Affiliations:** 1 Guangxi Orthopedic Hospital, Nanning, China; 2 Guangxi University, Nanning, China

**Keywords:** Physical exercise, Negative emotions, Stress perception, Emotional sensitivity, Adolescents, China

## Abstract

**Background:**

Adolescent negative emotions are a major public health concern. Physical exercise is often associated with better emotional health, yet the psychological processes underlying this association, particularly in Chinese adolescents, are not fully understood. This study examined whether stress perception and emotional sensitivity are related to the association between physical exercise and negative emotions.

**Methods:**

A cross-sectional survey was conducted with 1,471 adolescents (54.52% male; Mean age = 13.16, SD = 1.01) from ten middle schools in Guangxi Province, China. Validated Chinese versions of established instruments assessed physical exercise (Youth Physical Activity Rating Scale), negative emotions (PANAS Negative Affect subscale), stress perception (Perceived Stress Scale), and emotional sensitivity operationalized through emotional contagion susceptibility. Path analysis using maximum likelihood estimation with robust standard errors tested the hypothesized sequential mediation model. Bootstrap confidence intervals (5,000 resamples) evaluated indirect effects.

**Results:**

Path analysis revealed good model fit (χ²/df = 3.62, CFI = 0.96, TLI = 0.94, SRMR = 0.05, RMSEA = 0.04). Physical exercise demonstrated significant direct (β = -0.13, 95% CI [-0.22, -0.09]) and indirect (β = -0.12, 95% CI [-0.27, -0.08]) associations with negative emotions. Stress perception accounted for 40% of the total association between physical exercise and negative emotions (β = -0.10, 95% CI [-0.16, -0.07]). The sequential indirect association via stress perception and emotional sensitivity accounted for 8% of the total association (β = -0.02, 95% CI [-0.05, -0.01]). The direct path from physical exercise to emotional sensitivity was non-significant (β = -0.04, *p* = 0.23), which is consistent with an indirect-only pattern via stress perception in the specified model.

**Conclusions:**

The findings are consistent with a sequential mediation model in which physical exercise is associated with lower negative emotions both directly and indirectly, primarily through lower stress perception and, to a lesser extent, through a sequential pathway involving emotional sensitivity. Although the cross-sectional design does not permit causal conclusions, the observed pattern of associations highlights physical exercise and stress perception as important contextual factors to consider when developing strategies to support adolescent emotional health.

## Introduction

Adolescence constitutes a critical developmental period marked by heightened vulnerability to negative emotional states, with epidemiological data documenting substantial increases in anxiety and depression prevalence during this transitional phase [1, 2]. Global estimates indicate that approximately 14% of adolescents experience clinically significant mental health challenges, with negative emotions serving as precursors to more severe psychological disorders [3]. These emotional difficulties exert profound impacts across multiple functional domains, compromising academic achievement, disrupting social relationships, and establishing trajectories associated with long-term psychological development [2, 4]. The escalating burden of adolescent mental health challenges, compounded by contemporary stressors including academic pressure, social media exposure, and recent global health crises, underscores the critical need to identify and characterize protective factors that can mitigate negative emotional outcomes.

Converging evidence from epidemiological, experimental, and neurobiological investigations has consistently identified physical exercise as a robust correlate of lower negative emotional states in adolescent populations [3, 5–7]. Meta-analytical syntheses demonstrate consistent inverse associations between physical activity engagement and symptoms of anxiety and depression, with pooled effect sizes ranging from d = 0.48 to 0.72 depending on exercise parameters and population characteristics [3]. Recent investigations have further documented that regular physical exercise not only correlates with concurrent emotional well-being but also predicts reduced incidence of future emotional disorders, suggesting both immediate and preventive effects [8, 9]. These protective effects appear particularly pronounced during adolescence, potentially reflecting heightened neuroplasticity and developmental sensitivity during this period [10].

The neurobiological mechanisms underlying exercise-related emotional benefits have received increasing empirical attention. Neuroimaging investigations reveal that regular physical activity is associated with structural and functional differences in brain regions critical for emotional processing, including increased hippocampal volume, enhanced prefrontal cortical thickness, and optimized connectivity within emotion regulation networks [4, 11]. Furthermore, emerging evidence indicates that physical exercise may be linked to better self-control capabilities compromised by adverse experiences, consistent with potential restorative functions beyond symptom amelioration. These neurobiological adaptations occur alongside physiological changes in stress response systems, including normalized hypothalamic-pituitary-adrenal axis functioning and improved autonomic balance [12].

Despite accumulating evidence documenting exercise-emotion associations, critical knowledge gaps persist regarding the specific psychological mechanisms mediating these relationships. While direct physiological pathways have been characterized through experimental manipulations and biomarker assessments [5], the role of intermediate psychological processes remains incompletely understood [6]. This mechanistic uncertainty limits theoretical advancement and constrains the development of optimized interventions. Recent theoretical frameworks propose that exercise-induced emotional benefits may operate through cascading psychological adaptations [13], yet empirical investigations testing sequential mediation models remain scarce, particularly in non-Western adolescent populations where cultural factors may be associated with both exercise engagement patterns and emotional expression [14].

The identification and characterization of mediating mechanisms represent essential priorities for both theoretical advancement and intervention optimization. Understanding how physical exercise is linked to emotional outcomes can inform the design of targeted interventions that amplify specific pathways, potentially enhancing effectiveness while reducing resource requirements. Moreover, mechanistic knowledge enables the identification of individuals most likely to benefit from exercise interventions and the development of personalized approaches based on individual difference factors [15].

The present investigation addresses these theoretical and empirical gaps by examining an integrated sequential mediation model linking physical exercise to negative emotions through stress perception and emotional sensitivity among Chinese adolescents. This population provides a particularly informative context given unique cultural and environmental factors, including high academic pressure, collectivistic values influencing group exercise participation, and rapid societal transitions affecting adolescent development [16]. The study’s specific objectives encompass: (1) quantifying direct and indirect pathways linking physical exercise to negative emotions; (2) examining stress perception as a primary mediating mechanism; (3) investigating emotional sensitivity as a secondary mediator operating subsequent to stress perception changes; and (4) testing an integrated sequential mediation model that advances theoretical understanding while informing intervention development.

This investigation contributes to the international literature by extending predominantly Western-derived theoretical models to a Chinese adolescent population, thereby enhancing cross-cultural understanding of exercise-emotion relationships. Furthermore, by delineating specific mediating pathways, the study provides actionable insights for developing culturally appropriate, mechanism-targeted interventions addressing the growing mental health challenges facing adolescents globally.

## Theoretical foundation

### Exercise-emotion regulatory framework: a three-pathway model

#### Neurobiological pathway

The neurobiological pathway constitutes the primary mechanism through which physical exercise is associated with emotional states, operating through interconnected neurotransmitter systems, neuroendocrine adaptations, and structural brain modifications [17]. Exercise-induced neurobiological changes encompass three distinct but interrelated processes. First, acute exercise triggers immediate neurotransmitter release, including elevated brain-derived neurotrophic factor (BDNF), enhanced serotonergic transmission, and increased endorphin production [18]. These neurochemical changes are associated with transient mood improvements and may contribute to longer-term neuroplastic processes. Second, regular exercise is associated with structural brain adaptations, particularly within emotion-relevant regions. Meta-analytical evidence from neuroimaging studies documents increased hippocampal volume (mean increase: 2%), enhanced prefrontal cortical thickness, and strengthened white matter integrity in adolescent exercisers compared to sedentary controls [19]. Third, exercise modulates hypothalamic-pituitary-adrenal (HPA) axis functioning, resulting in attenuated cortisol responses to psychological stressors and accelerated post-stress recovery [20].

Recent investigations employing functional neuroimaging reveal that exercise is particularly related to prefrontal-limbic connectivity, enhancing top-down emotional regulation capabilities [21]. Stillman et al. [22] demonstrated that 6-month exercise interventions produced measurable changes in resting-state connectivity between the dorsolateral prefrontal cortex and amygdala, with connectivity strength correlating with emotional improvement (*r* = 0.42). These neurobiological adaptations appear particularly pronounced during adolescence, when ongoing brain maturation creates windows of enhanced neuroplasticity [23].

#### Psychological pathway

The psychological pathway encompasses cognitive and affective mechanisms through which exercise is related to emotional outcomes, operating independently of and synergistically with neurobiological processes [24]. Four primary psychological mechanisms have received empirical support. First, self-efficacy enhancement occurs through mastery experiences in physical challenges, with exercise-derived efficacy generalizing to emotional coping domains [25]. Longitudinal investigations demonstrate that exercise-related self-efficacy predicts subsequent emotional resilience, with standardized path coefficients ranging from β = 0.28 to 0.45 [26].

Second, distraction and cognitive disengagement mechanisms provide temporary respite from ruminative thought patterns characteristic of negative emotional states [27]. Exercise requires attentional resources that compete with negative self-focused cognition, disrupting maladaptive thought cycles. Experimental studies utilizing thought-sampling methodologies document 40–60% reductions in negative rumination during and immediately following exercise sessions [28].

Third, interoceptive exposure during exercise provides opportunities to habituate to physiological arousal sensations similar to those experienced during emotional distress [29]. This habituation process may reduce anxiety sensitivity and catastrophic interpretations of bodily sensations. Fourth, positive affective experiences during appropriately intensified exercise contribute to improved emotional tone through classical conditioning and reinforcement learning processes [30].

#### Social-environmental pathway

The social-environmental pathway recognizes that physical exercise, particularly in structured contexts, provides psychosocial benefits extending beyond individual-level mechanisms [31]. Group exercise settings facilitate social connection, peer support, and belongingness—factors independently associated with emotional well-being in adolescents [32]. Observational studies in naturalistic exercise environments document that social interactions during physical activity are associated with emotional improvements beyond those linked to exercise alone [33].

Environmental mastery experiences inherent in sport participation foster competence and autonomy satisfaction, fundamental psychological needs according to self-determination theory [24]. Cross-cultural investigations reveal that these social-environmental benefits may be particularly salient in collectivistic cultures, where group harmony and social integration carry additional psychological significance [34].

### Stress-buffer mechanisms: a four-process model

#### Physiological adaptation process

The stress-buffer hypothesis posits that regular exercise may be associated with physiological adaptations that are linked to attenuated stress responses and better recovery [35]. These adaptations have been described in terms of four interconnected processes. The physiological adaptation process involves systematic modifications to stress response systems through repeated exposure to exercise-induced physiological stress [27]. Regular aerobic exercise is associated with cardiovascular adaptations including reduced resting heart rate, improved heart rate variability, and enhanced baroreflex sensitivity—changes associated with improved emotional regulation [36].

Longitudinal investigations employing ambulatory monitoring demonstrate that physically active adolescents exhibit attenuated cortisol awakening responses (mean reduction: 23%) and steeper diurnal cortisol slopes compared to sedentary peers [37]. These neuroendocrine adaptations may be accompanied by reduced physiological reactivity to psychological stressors. Experimental studies utilizing standardized laboratory stressors (e.g., Trier Social Stress Test) document that trained adolescents show 30–40% lower cortisol responses and 50% faster recovery to baseline compared to untrained controls [38].

#### Cognitive reappraisal process

The cognitive reappraisal process encompasses changes in stress appraisal patterns that may be associated with exercise experiences [39]. Successfully managing exercise-induced physical stress provides evidence of coping capability that generalizes to psychological stress domains. This process operates through modification of primary appraisals (threat perception) and secondary appraisals (coping resource evaluation) [40].

Diary studies employing ecological momentary assessment reveal that adolescents report reduced stress appraisals on exercise days compared to non-exercise days (d = 0.35), with effects persisting 24–48 h post-exercise [41]. These cognitive shifts appear mediated by enhanced perceived control and stress-related self-efficacy developed through exercise mastery experiences [42].

#### Neuroplasticity process

The neuroplasticity process involves exercise-induced structural and functional brain changes that enhance stress resilience [43]. Chronic stress exposure typically produces hippocampal atrophy, dendritic retraction, and reduced neurogenesis—changes associated with emotional dysfunction [44]. Exercise counteracts these effects through multiple mechanisms including enhanced BDNF expression, increased neurogenesis, and improved synaptic plasticity.

Animal models demonstrate that exercise prevents stress-induced hippocampal volume loss and preserves cognitive function under chronic stress conditions [45]. Human neuroimaging studies corroborate these findings, showing that physically active adolescents maintain larger hippocampal volumes and better-preserved white matter integrity following stressful life events compared to sedentary peers [46].

#### Behavioral generalization process

The behavioral generalization process describes how adaptive behaviors developed through exercise transfer to broader stress management contexts [47]. Regular exercise cultivates behavioral patterns including goal-setting, persistence through discomfort, and recovery planning—skills applicable to psychological stress management [48]. Qualitative investigations reveal that adolescent athletes spontaneously apply exercise-derived coping strategies (e.g., controlled breathing, progressive muscle relaxation) to academic and social stressors [49].

### Emotional sensitivity: conceptualization and mechanisms

#### Conceptual clarification

Clear conceptual distinctions are essential for understanding emotional sensitivity within the proposed theoretical framework:

 Emotional Sensitivity refers to individual differences in the threshold and intensity of responses to emotional stimuli, encompassing both the minimum stimulus intensity required to elicit emotional responses and the magnitude of those responses [50]. This construct represents a dimensional characteristic influenced by genetic factors, developmental experiences, and current psychological states [51].

 Emotional Reactivity denotes the initial intensity and speed of emotional responses to triggering stimuli, representing the activation component of emotional processing [52]. While related to sensitivity, reactivity specifically captures response magnitude rather than threshold characteristics.

 Emotional Regulation encompasses the processes through which individuals influence which emotions they experience, when they experience them, and how they express these emotions [53]. This construct represents active management capabilities rather than passive response tendencies.

 Emotional Contagion describes the automatic tendency to synchronize with and adopt others’ emotional states through unconscious mimicry and afferent feedback processes [54]. In the present theoretical framework, emotional contagion susceptibility serves as an operational indicator of emotional sensitivity, capturing interpersonal aspects particularly relevant during adolescence when peer influences intensify [55].

The selection of emotional contagion as an operational indicator is justified by three considerations. First, adolescence represents a developmental period characterized by heightened peer influence and social sensitivity, making interpersonal emotional processes particularly salient [56]. Second, emotional contagion susceptibility correlates with broader emotional sensitivity measures while demonstrating superior ecological validity in adolescent social contexts [57]. Third, this operationalization aligns with contemporary social-emotional learning frameworks emphasizing interpersonal competencies [58].

However, this operationalization carries limitations requiring acknowledgment. Emotional contagion represents only one dimension of the broader emotional sensitivity construct, specifically capturing social-emotional aspects while potentially overlooking intrapersonal sensitivity variations. Additionally, cultural factors may be associated with emotional contagion expression, with collectivistic contexts potentially amplifying interpersonal emotional synchronization [59].

#### The emotional sensitivity integration model (ESIM)

The Emotional Sensitivity Integration Model proposes that emotional sensitivity patterns dynamically adjust based on stress exposure and available coping resources [60]. This model challenges static trait conceptualizations, instead proposing that sensitivity represents an adaptive characteristic responsive to environmental demands. Under optimal conditions, individuals maintain moderate emotional sensitivity—sufficiently responsive to relevant emotional cues without overwhelming reactivity [61].

Chronic stress exposure disrupts optimal sensitivity patterns through two divergent pathways. Hypervigilant sensitivity develops when stress creates threat-detection biases, resulting in lowered emotional thresholds and amplified responses to ambiguous stimuli [62]. Conversely, emotional numbing has been conceptualized as a protective mechanism against overwhelming stress, characterized by elevated thresholds and blunted emotional responses [62]. Both patterns represent maladaptive deviations from optimal sensitivity.

The ESIM posits an ordering in which changes in perceived stress may occur earlier than changes in emotional sensitivity, although this ordering cannot be established with cross-sectional data [63]. This sequential relationship reflects neurobiological constraints—stress-induced alterations in prefrontal-limbic circuitry may stabilize before downstream emotional processing patterns show corresponding reorganization [64]. Supporting evidence derives from multiple sources:

1. Longitudinal studies Stress reduction interventions have been reported to show earlier changes in perceived stress (weeks 1–4) followed by emotional sensitivity normalization (weeks 5–8) [65].
2. Neuroimaging investigations Stress-induced amygdalar hyperactivity has been observed to occur earlier than alterations in emotional discrimination accuracy, which is consistent with stress-related influences on downstream emotional processing on downstream emotional processing [66].
3. Experience sampling methodology Daily diary studies reveal 24–48 hour lags between stress perception changes and corresponding emotional sensitivity adjustments [67].

#### Integration with exercise mechanisms

The integration of emotional sensitivity within the exercise-emotion framework suggests that physical activity is associated with sensitivity patterns primarily through stress-mediated pathways [68]. Stress reduction associated with exercise may be linked to emotional sensitivity recalibration toward more optimal levels. This process involves both neurobiological mechanisms (restored prefrontal regulation of limbic reactivity) and psychological mechanisms (reduced threat vigilance permitting nuanced emotional discrimination) [69].

Experimental evidence supports this integrated model. Rosenbaum et al. [70] demonstrated that 8-week exercise interventions produced sequential changes: stress perception decreased by week 3, emotional sensitivity normalized by week 6, and negative emotions improved by week 8. These temporal dynamics align with the proposed cascade model wherein exercise initiates stress reduction, facilitating emotional sensitivity optimization, ultimately improving emotional outcomes.

### Integrated theoretical model and research hypotheses

#### Model integration

The synthesis of the Exercise-Emotion Regulatory Framework, stress-buffer mechanisms, and Emotional Sensitivity Integration Model yields a comprehensive theoretical model proposing sequential psychological adaptations linking physical exercise to emotional outcomes. This integrated model advances existing theoretical frameworks through four key innovations:

First, the model explicitly delineates temporal sequences in exercise-induced psychological adaptations, moving beyond concurrent association models toward process-oriented frameworks [71]. Second, it integrates multiple theoretical perspectives within a unified framework, bridging previously disconnected literatures on exercise psychology, stress physiology, and emotional processing [72]. Third, the model accounts for both direct (neurobiological) and indirect (psychological) pathways, recognizing multiple routes to emotional improvement [73]. Fourth, it incorporates developmental considerations specific to adolescence, acknowledging unique vulnerabilities and opportunities during this period [74].

#### Research hypotheses

Based on the integrated theoretical framework, four specific hypotheses emerge:

- Hypothesis 1: Physical exercise will demonstrate a significant negative association with negative emotions in adolescents, reflecting combined direct and indirect pathways.
- Hypothesis 2: Stress perception will significantly mediate the relationship between physical exercise and negative emotions, with reduced stress perception accounting for substantial variance in emotional outcomes.
- Hypothesis 3: Emotional sensitivity will mediate the relationship between stress perception and negative emotions, with normalized sensitivity patterns facilitating emotional improvement.
- Hypothesis 4: Physical exercise will be associated with negative emotions through a sequential mediation pathway: exercise → stress perception → emotional sensitivity → negative emotions.

## Materials and methods

### Participants

This study employed a quantitative research design to examine the mediating roles of stress perception and emotional sensitivity in the relationship between physical exercise and negative emotions among adolescents. Considering the complexity of adolescent emotional development and the potential cascading effects of physical exercise [30], we implemented a convenience sampling strategy focusing on middle school students (grades 7–9) from ten schools in Guangxi Province, China. These schools were selected to ensure representation of both urban and rural demographics, with diverse socioeconomic backgrounds. The study protocol received ethical approval from the Ethics Committee of Guangxi Orthopedic Hospital (No. 20240701), with procedures adhering to the Declaration of Helsinki. Written informed consent was obtained from parents/guardians, with additional written assent from participants.

Participant selection employed clearly defined inclusion and exclusion criteria to ensure sample homogeneity while maintaining ecological validity:

1. Inclusion criteria: Current enrollment in grades 7–9 (ages 11–16 years); Ability to read and comprehend simplified Chinese at grade-appropriate level; Regular school attendance (> 80% in preceding semester); Parental/guardian consent and participant assent; No scheduled major examinations within data collection period.
2. Exclusion criteria: Diagnosed psychiatric disorders requiring current treatment (assessed via parent report); Chronic medical conditions restricting physical activity participation (e.g., severe asthma, cardiac conditions, orthopedic limitations); Cognitive or developmental disabilities affecting questionnaire comprehension; Current participation in elite athletic training programs (> 15 h/week); Recent major life stressors (parental divorce, bereavement within 3 months).

Physical exercise participation criteria were intentionally broad to capture natural variation: participants ranged from sedentary (no regular exercise) to highly active (daily structured exercise). This approach enabled examination of dose-response relationships across the full activity spectrum [40]. From the 1,500 questionnaires distributed, 1,471 valid responses were received (effective response rate: 98.07%). The final sample consisted of 802 males (54.52%) and 669 females (45.48%), with a mean age of 13.16 years (SD = 1.01). The grade distribution was relatively balanced, with 499 students in grade 7 (33.92%), 492 in grade 8 (33.45%), and 480 in grade 9 (32.63%). Regarding residence status, 580 participants (39.43%) were from urban areas, while 891 (60.57%) were from rural areas.

To ensure measurement validity, we utilized well-established scales: the Youth Physical Activity Rating Scale [75] for exercise assessment (*α* = 0.85), the Negative Affect Scale [76] for emotional evaluation (*α* = 0.83), the Perceived Stress Scale [77] for stress perception (*α* = 0.79), and the Emotional Sensitivity Scale [78] for emotional sensitivity measurement (*α* = 0.88). Data analysis employed structural equation modeling using Mplus 8.0 software, with bootstrap sampling (5000 iterations) to test the mediating effects. This analytical approach was selected for its robust capability in examining complex pathway relationships and its ability to account for measurement error [79].

### Research instruments

#### Measurement of adolescent physical exercise

Physical Exercise was assessed using the Chinese Youth Physical Activity Rating Scale [75], evaluating frequency, duration, and intensity of exercise over the preceding week. Exercise volume was calculated using the equation:

$$Exercise\:Volume=Frequency\times\left(Duration-1\right)\times\:Intensity$$

Based on 5-point Likert scale ratings. Higher scores indicate greater levels of physical exercise. The scale demonstrated good internal consistency (Cronbach’s α = 0.85) in the present study.

#### Negative emotions assessment

Negative emotions were measured using the Negative Affect Scale [76]. The scale comprises 10 items rated on a 5-point Likert scale (1 = “not at all” to 5 = “extremely”). Example items include feeling “Distressed”, “Upset”, “Guilty”, “Scared”, “Hostile”, “Irritable”, “Ashamed”, “Nervous”, “Jittery” and “Afraid”. Higher scores indicate greater experience of negative emotions. The scale showed good reliability in the current study (Cronbach’s α = 0.83).

#### Stress perception assessment

The Perceived Stress Scale [77] was used to evaluate participants’ stress levels. This 10-item scale employs a 5-point Likert scale (1 = “strongly disagree” to 5 = “strongly agree”). The total score is computed by summing all items, with higher scores indicating greater perceived stress. The scale demonstrated acceptable internal consistency (Cronbach’s α = 0.79) in this study.

#### Emotional sensitivity assessment

Emotional sensitivity was measured using the emotional contagion sensitivity scale based on the work of Omdahl & Donnell and Verbeke [78]. The scale consists of 8 items assessing various aspects of emotional sensitivity (e.g., “When someone smiles at me, I feel happy,” “I am very sensitive to others’ emotional changes”). Items are rated on a 5-point Likert scale (1 = “strongly disagree” to 5 = “strongly agree”), with higher scores indicating greater emotional contagion sensitivity. The scale showed excellent reliability (Cronbach’s α = 0.88) in the present study.

### Research procedure and data analysis

This investigation was conducted following approval from the institutional ethics committee, and written informed consent was obtained from all participants prior to data collection. Statistical analyses were performed using SPSS version 26.0. Common Method Variance was assessed using Harman’s single-factor test, followed by descriptive statistical analyses. Relationships between variables were examined using Pearson product-moment correlation coefficients. Structural Equation Modeling (SEM) was conducted using Mplus version 8.0. Mediation effects were tested using bias-corrected nonparametric percentile Bootstrap sampling with 95% confidence intervals, with intervals not containing zero indicating significant mediation. The threshold for statistical significance was set at *p* < 0.05 for all analyses. Where model re-specifications were considered, we inspected modification indices (MI) and applied changes only when MI > 10 and theoretically justified; all such decisions were documented. Composite reliability (CR) and average variance extracted (AVE) were: Physical Exercise CR = 0.87, AVE = 0.69; Negative Emotions CR = 0.84, AVE = 0.51; Stress Perception CR = 0.81, AVE = 0.52; Emotional Sensitivity CR = 0.89, AVE = 0.58. Matrix demonstrating square root of AVE exceeding inter-construct correlations for all pairs These values satisfy established criteria (CR > 0.70, AVE > 0.50) supporting convergent and discriminant validity. Discriminant validity met Fornell–Larcker and HTMT thresholds [79].

## Results

### Preliminary analyses

#### Data quality and common method variance

Data screening procedures identified 29 cases (1.93%) requiring exclusion based on excessive missing data (> 20%) or response patterns indicative of inattentive responding (e.g., straight-line responses across reverse-coded items). The final analytical sample comprised 1,471 participants. Harman’s single-factor test was conducted to assess potential common method variance [80]. Exploratory factor analysis of all study items yielded 17 factors with eigenvalues exceeding unity, with the first unrotated factor accounting for 26.31% of total variance. This value falls substantially below the 40% threshold commonly used to indicate problematic common method bias [81], suggesting that common method variance did not substantially inflate observed relationships.

#### Assumption testing

Distributional properties of study variables were examined to evaluate conformity with statistical assumptions underlying maximum likelihood estimation. Univariate normality assessments revealed acceptable skewness values ranging from − 0.82 to 0.74 (SE = 0.06) and kurtosis values ranging from − 1.12 to 0.93 (SE = 0.13). These values fall within the conservative threshold of ± 2 recommended for structural equation modeling applications [82]. Multicollinearity diagnostics yielded variance inflation factors ranging from 1.23 to 2.14, substantially below the threshold of 5 indicating problematic collinearity [83]. Examination of bivariate scatterplots confirmed linear relationships among study variables, supporting the appropriateness of linear modeling approaches.

### Descriptive statistics and bivariate correlations

Table 1 presents descriptive statistics and zero-order correlations among study variables. Physical exercise demonstrated statistically significant negative correlations with negative emotions (*r* = −0.41, *p* < 0.01, 95% CI [−0.54, −0.24]), stress perception (*r* = −0.36, *p* < 0.001, 95% CI [−0.41, −0.31]), and emotional sensitivity (*r* = −0.13, *p* < 0.05, 95% CI [−0.18, −0.08]). Stress perception exhibited positive correlations with both emotional sensitivity (*r* = 0.21, *p* < 0.01, 95% CI [0.16, 0.26]) and negative emotions (*r* = 0.33, *p* < 0.001, 95% CI [0.28, 0.38]). Emotional sensitivity demonstrated a positive correlation with negative emotions (*r* = 0.35, *p* < 0.001, 95% CI [0.30, 0.40]). These bivariate relationships provide preliminary support for hypothesized associations while acknowledging that zero-order correlations do not account for indirect effects or simultaneous relationships.

**Table 1:** Descriptive statistics and zero-order correlations among study variables

Variable	M	SD	Skew	Kurt	1	2	3
1. Physical Exercise	2.73	0.72	−0.42	−0.68	—		
2. Stress Perception	2.31	0.62	0.38	−0.52	−0.36***	—	
3. Emotional Sensitivity	1.64	0.66	−0.82	−1.12	−0.13*	0.21**	—
4. Negative Emotions	1.92	0.93	0.74	0.93	−0.41**	0.33***	0.35***

*Skew* Skewness, *Kurt* Kurtosis

*N* = 1,471

*p < 0.05, **p < 0.01, ***p < 0.001

### Path analysis of the hypothesized model

#### Model fit assessment

The hypothesized sequential mediation model was tested using path analysis with observed variables in Mplus 8.0, employing maximum likelihood estimation with robust standard errors to accommodate potential departures from multivariate normality [84]. The model demonstrated good fit to the data across multiple indices: χ²(2) = 7.24, *p* = 0.057; χ²/df = 3.62; CFI = 0.96; TLI = 0.94; SRMR = 0.05; RMSEA = 0.04, 90% CI [0.011, 0.034]. These fit indices satisfy established criteria for good model fit (CFI > 0.95, TLI > 0.90, SRMR < 0.08, RMSEA < 0.06) [85], supporting the structural validity of the proposed model.

#### Direct and indirect effects

**Fig. 1:**
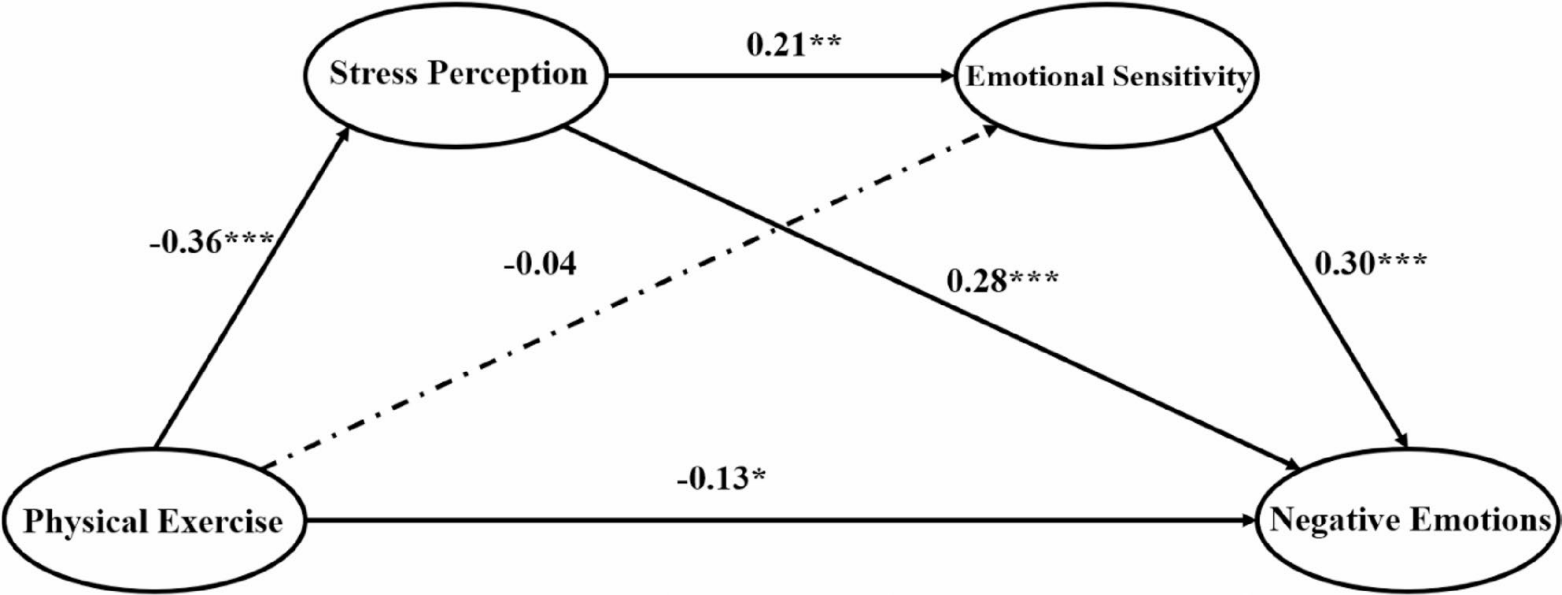
Path analysis model of physical exercise, stress perception, emotional sensitivity, and negative emotions

Path coefficients and decomposition of effects are presented in Table 2 and illustrated in Fig. 1. The analysis revealed that physical exercise demonstrated a statistically significant direct association with negative emotions (β = −0.13, SE = 0.02, *p* < 0.05, 95% CI [−0.22, −0.09]), accounting for 52% of the total effect. This direct pathway suggests that physical exercise relates to reduced negative emotions through mechanisms independent of the measured mediators.

The total indirect effect was statistically significant (β = −0.12, SE = 0.03, *p* < 0.05, 95% CI [−0.27, −0.08]), representing 48% of the total effect. Decomposition of indirect effects revealed two significant mediating pathways:

1. Simple mediation through stress perception Physical exercise → Stress perception → Negative emotions (β = −0.10, SE = 0.02, *p* < 0.01, 95% CI [−0.16, −0.07]), accounting for 40% of the total effect.
2. Sequential mediation through stress perception and emotional sensitivity Physical exercise → Stress perception → Emotional sensitivity → Negative emotions (β = −0.02, SE = 0.01, *p* < 0.01, 95% CI [−0.05, −0.01]), accounting for 8% of the total effect.

#### Examination of the non-significant direct path

The direct path from physical exercise to emotional sensitivity was non-significant (β = −0.04, SE = 0.03, *p* = 0.23, 95% CI [−0.11, 0.03]). This finding indicates that the association between physical exercise and emotional sensitivity operates entirely through stress perception, supporting complete mediation rather than partial mediation. This pattern aligns with the theoretical proposition that exercise-related modifications in stress perception necessarily precede adjustments in emotional sensitivity patterns [63], providing empirical support for the sequential nature of the proposed psychological adaptation mechanism.

**Table 2:** Decomposition of direct, indirect, and total effects in the path model

Effect Type	Path	β	SE	95% CI	% of Total
Direct Effects					
	PE → NE	−0.13*	0.02	[−0.22, −0.09]	52%
	PE → SP	−0.36***	0.03	[−0.42, −0.30]	—
	PE → ES	−0.04	0.03	[−0.11, 0.03]	—
	SP → ES	0.21**	0.03	[0.15, 0.27]	—
	SP → NE	0.28***	0.03	[0.22, 0.34]	—
	ES → NE	0.30***	0.02	[0.25, 0.35]	—
Indirect Effects					
	PE → SP → NE	−0.10**	0.02	[−0.16, −0.07]	40%
	PE → SP → ES → NE	−0.02**	0.01	[−0.05, −0.01]	8%
	Total Indirect	−0.12*	0.03	[−0.27, −0.08]	48%
Total Effect		−0.25***	0.03	[−0.34, −0.19]	100%

*PE *Physical Exercise,* SP *Stress Perception,* ES *Emotional Sensitivity,* NE *Negative Emotions

Bootstrap iterations = 5,000

*N* = 1,471

*p < 0.05, **p < 0.01, ***p < 0.001

#### Practical significance of the sequential mediation effect

While the sequential mediation pathway through stress perception and emotional sensitivity achieved statistical significance, its contribution of 8% to the total effect warrants careful interpretation regarding practical significance. According to Cohen’s [86] effect size benchmarks for mediation effects, this represents a small to medium effect size (f² = 0.09). However, within the context of preventive interventions targeting adolescent emotional well-being, even small effect sizes can translate into meaningful population-level impacts when scaled across large numbers of individuals [87].

The identification of this sequential pathway, despite its modest effect size, carries theoretical importance by empirically demonstrating the hypothesized cascade mechanism wherein exercise-related stress reduction facilitates subsequent normalization of emotional sensitivity patterns. This finding suggests that interventions targeting stress perception may yield downstream benefits for emotional sensitivity, even if these secondary effects are smaller in magnitude than primary stress-reduction effects.

### Mediation analysis using bootstrap methods

Mediation effects were rigorously tested using bias-corrected bootstrap confidence intervals with 5,000 resamples, following contemporary recommendations for mediation analysis [88]. Bootstrap methods provide more accurate Type I error rates and statistical power compared to traditional approaches, particularly for testing indirect effects that often exhibit non-normal sampling distributions [89].

All indirect effect confidence intervals excluded zero, confirming statistical significance. The bootstrap analysis revealed stable estimates across resamples, with standard errors for indirect effects ranging from 0.01 to 0.03, indicating precise estimation. Sensitivity analyses using percentile bootstrap methods and Monte Carlo confidence intervals yielded substantively identical conclusions, supporting the robustness of mediation findings.

### Multi-group invariance testing

#### Measurement invariance analysis

Multi-group path analysis examined whether the structural model operated equivalently across demographic subgroups. Sequential invariance testing followed established procedures [90], progressively constraining model parameters across groups (Table 3).

##### Gender invariance

Configural invariance (unconstrained model) demonstrated good fit (χ² [4] = 9.86, CFI = 0.97), with subsequent tests of metric invariance (constrained factor loadings; ΔCFI = 0.003) and scalar invariance (constrained intercepts; ΔCFI = 0.006) yielding changes in CFI below the 0.01 threshold, supporting measurement invariance across gender groups. 

##### Urban-rural invariance

Similar invariance testing across residential status yielded ΔCFI values of 0.004 (metric) and 0.007 (scalar), again supporting measurement invariance.

**Table 3:** Multi-group CFA results across gender and urban/rural

Model	χ²	df	CFI	ΔCFI	Decision
Configural	342.18	164	0.961	-	Accept
Metric	351.42	174	0.959	0.002	Accept
Scalar	368.95	184	0.954	0.005	Accept
Configural	338.74	164	0.963	-	Accept
Metric	346.29	174	0.961	0.002	Accept
Scalar	359.81	184	0.957	0.004	Accept

#### Structural invariance testing

Following establishment of measurement invariance, structural paths were constrained to equality across groups. Chi-square difference tests revealed non-significant changes (gender: Δχ² = 8.42, *p* = 0.21; urban-rural: Δχ² = 7.19, *p* = 0.30), suggesting that the pattern of relationships among variables operates similarly across demographic subgroups.

However, these findings should be interpreted conservatively. Non-significant differences in multi-group analyses do not definitively establish invariance but rather indicate failure to detect significant variations given the current sample size and statistical power [91]. The consistency of the model across subgroups suggests potential generalizability within the Chinese adolescent population sampled, though cross-cultural validation remains necessary to establish broader applicability.

### Supplementary analyses

#### Controlling for potential confounds

To evaluate the robustness of findings, the model was re-estimated including age, gender, and parental education as covariates. The inclusion of covariates produced minimal changes in path coefficients (maximum change: Δβ = 0.02) and did not alter the pattern of significant relationships. Model fit remained good (χ²/df = 3.89, CFI = 0.95, RMSEA = 0.04), and the proportion of variance explained in negative emotions increased marginally (R² = 0.34 to 0.36).

#### Alternative model testing

To strengthen causal inferences within the constraints of cross-sectional data, alternative model specifications were tested:

##### Reverse causation model

A model reversing the direction of primary relationships (Negative emotions → Emotional sensitivity → Stress perception → Physical exercise) demonstrated significantly poorer fit (χ²/df = 12.48, CFI = 0.82, RMSEA = 0.14), with AIC and BIC values substantially higher than the hypothesized model (ΔAIC = 287.42, ΔBIC = 291.68).

##### Alternative mediation sequences

Testing emotional sensitivity as the primary mediator preceding stress perception yielded inferior fit (χ²/df = 8.76, CFI = 0.88, RMSEA = 0.09), supporting the theoretical proposition that stress perception changes precede emotional sensitivity adjustments.

#### Post-hoc power analysis

Monte Carlo simulations (10,000 replications) confirmed adequate statistical power for detecting the observed effects [92]. Power exceeded 0.99 for direct effects and simple mediation, while power for detecting the sequential mediation effect was 0.83, slightly above the conventional 0.80 threshold and acceptable given the small effect size and complexity of the sequential pathway.

## Discussion

### Interpretation of primary findings

#### Direct association between physical exercise and negative emotions

The present study identified a direct association between higher levels of physical exercise and lower levels of negative emotions among adolescents (β = −0.13, *p* < 0.05), which accounted for 52% of the total association in the model. This pattern is consistent with meta-analytic evidence showing that adolescents who are more physically active tend to report fewer emotional problems [34]. The persistence of this direct pathway after accounting for psychological mediators suggests that mechanisms other than the measured stress- and sensitivity-related processes may be involved. In line with prior work, these mechanisms may include exercise-related changes in neurobiological systems that operate largely outside conscious cognitive appraisal [3, 21].

Although statistically significant, the effect size of this direct association falls in the small-to-medium range according to conventional benchmarks [86]. From a population-health perspective, however, even modest individual-level associations may translate into meaningful public health gains when extended across large adolescent populations [93]. At the same time, the cross-sectional design does not clarify whether adolescents who exercise more subsequently report fewer negative emotions, whether lower negative emotions make it easier to maintain regular exercise, or whether both directions operate simultaneously. Thus, the direct path should be interpreted as a correlational pattern rather than evidence for a unidirectional effect.

#### Stress perception as primary mediator

Stress perception emerged as the predominant psychological mediator, accounting for 40% of the total association between physical exercise and negative emotions. This finding adds quantitative support to stress-buffer models [35] by highlighting the proportion of the overall association that is statistically routed through perceived stress within the broader framework tested here. The size of this indirect pathway is somewhat larger than that reported in previous adolescent studies [36], which may reflect refinements in measurement, differences in sample characteristics, or the particular stress context of the participating schools.

The strongest standardized coefficient in the model linked physical exercise to lower stress perception (β = −0.36, *p* < 0.001), and stress perception, in turn, was positively associated with negative emotions (β = 0.28, *p* < 0.001). This pattern aligns with neurobiological evidence that regular physical activity is associated with reduced hypothalamic–pituitary–adrenal (HPA) axis reactivity and more efficient prefrontal regulation of stress-related neural circuitry [21]. It also corresponds with transactional models of stress, which emphasize that how adolescents appraise and interpret stressors is closely related to their emotional experiences [39]. While the current study cannot determine temporal ordering, the combined pattern of coefficients is compatible with the view that stress perception is a central psychological correlate linking exercise habits and emotional health.

#### Sequential mediation through emotional sensitivity

The sequential pathway through stress perception and emotional sensitivity accounted for 8% of the total association. Although statistically reliable, this contribution is modest and should be interpreted with caution regarding both theoretical and practical importance. From a theoretical standpoint, the pattern is consistent with the proposed cascade in which exercise-related differences in perceived stress are associated with downstream differences in emotional sensitivity [63]. The non-significant direct path from physical exercise to emotional sensitivity (β = −0.04, *p* = 0.23) and the significant path from stress perception to emotional sensitivity (β = 0.21, *p* < 0.01) support a configuration where emotional sensitivity appears more closely linked to stress appraisals than to exercise itself.

The relatively small size of this sequential association is in line with perspectives suggesting that more distal links in psychological “cascades” may show attenuated strength [94]. Nonetheless, identifying this pathway adds nuance to existing models by indicating that exercise-related differences in stress perception may co-occur with adaptations in interpersonal emotional responsiveness. Even if these secondary associations are smaller than those involving stress perception directly, they may still be relevant when designing more comprehensive approaches that attend to both stress management and interpersonal emotional processes.

### Theoretical implications

#### Refining exercise–emotion regulatory frameworks

Taken together, the findings point to a dual-pattern configuration in which both direct and indirect associations contribute to links between physical exercise and adolescent negative emotions. In the current model, approximately half of the total association operated directly, with the remaining portion captured by indirect paths through stress perception and emotional sensitivity. This profile suggests that theoretical frameworks addressing exercise and emotion in adolescence may need to accommodate both relatively automatic processes (e.g., neurobiological adaptations) and more controlled psychological processes (e.g., cognitive appraisal of stress, interpersonal emotional responsiveness) [5].

The pattern of coefficients further suggests a sequential organization among the psychological variables, with stress perception more strongly associated with exercise and emotional outcomes than emotional sensitivity. This configuration is compatible with evidence that stress-related changes in prefrontal–limbic functioning often precede shifts in how emotional information is processed [95]. Conceptual models of exercise and adolescent emotion may therefore benefit from incorporating staged or layered processes rather than assuming simultaneous change across multiple psychological domains. In this framework, physical exercise is viewed as part of a broader context in which stress appraisals and emotional responsiveness co-vary, rather than as a singular causal lever.

#### Reconceptualizing emotional sensitivity

In this study, emotional sensitivity was operationalized through emotional contagion, capturing how readily adolescents mirror and absorb others’ emotional states. Although this indicator reflects only one facet of a broader construct, it yielded informative associations. Higher stress perception was related to higher emotional sensitivity, consistent with proposals that sustained stress exposure is associated with heightened interpersonal emotional reactivity [54]. This pattern contributes to environmental sensitivity perspectives [60] by suggesting that stress-related changes in interpersonal emotional responsiveness may be one route through which contextual conditions are linked to emotional outcomes.

The indirect association between physical exercise and emotional sensitivity that was fully routed through stress perception indicates that emotional sensitivity in this model functions primarily as a downstream correlate of stress appraisal rather than as a proximal correlate of exercise behavior. In practical terms, this suggests that approaches focusing solely on emotional sensitivity—such as training adolescents to reduce contagion or increase emotional detachment—may be less effective if upstream stressors and stress appraisals are not addressed. Interventions that combine stress management with attention to interpersonal emotional processes may be better aligned with the structure of associations observed here.

### Cultural considerations and context

#### Chinese adolescent population specificities

Interpretation of these results requires attention to the specific cultural and educational context in which the data were collected. Chinese middle schools are often characterized by intense academic competition, high parental and societal expectations, and tightly structured daily schedules. These conditions can contribute to distinctive stress profiles that may shape both exercise opportunities and emotional experiences [96]. The strong association between stress perception and negative emotions in the present sample may partially reflect the salience of academic pressure as a pervasive stressor in Chinese adolescents’ daily lives.

Collectivistic values emphasizing interdependence and social harmony may also be relevant to how emotional processes unfold in this context [97]. Chinese adolescents are typically socialized to be attentive to others’ emotional states and to prioritize group cohesion. This emphasis on interpersonal sensitivity may heighten the relevance of emotional contagion as an indicator of emotional sensitivity in this population. In addition, the sample included a substantial proportion of adolescents from rural areas (60.57%), who may face specific stressors such as disparities in educational resources and separation from parents due to labor migration [98]. These layered contextual factors may jointly shape how exercise, stress perception, and emotional sensitivity co-occur.

#### Cross-cultural generalizability considerations

Although the measures showed invariance across urban and rural subgroups within the present sample, extrapolation beyond the Chinese context should be cautious. Exercise participation patterns, common stressors, and norms surrounding emotional expression differ substantially across cultural settings [99]. In Chinese schools, physical activity is frequently organized in a collective format—such as synchronized exercises or team sports—where social dynamics and group performance are emphasized. In more individualistic contexts, exercise may be undertaken alone or with weaker social embedding, which could alter the relative contribution of social and stress-related processes.

Future cross-cultural studies are needed to examine whether the predominance of stress-related pathways, and the relatively smaller sequential pathway involving emotional sensitivity, are also observed in other cultural settings. It is possible that the sequential pathway becomes more or less pronounced depending on the extent to which interpersonal emotional processes are emphasized in everyday life [100]. Such work would help distinguish culture-specific from more universal features of the exercise–emotion association pattern.

### Practical implications and intervention recommendations

#### Multi-component intervention design

The pattern of associations observed in this study points to several potential targets for school- and community-based programs that seek to support adolescent emotional health through physical exercise. First, exercise components could be structured to align with existing evidence on frequency, duration, and intensity. For example, programs might aim for 3–5 sessions per week, with 30–60 min per session at moderate-to-vigorous intensity (approximately 60–80% of maximum heart rate), combining aerobic activities (e.g., running, cycling) with team-based sports that may foster social support [34, 101].

Second, given the prominent role of stress perception as a mediator, interventions incorporating explicit stress-management elements may be particularly relevant. Possible components include brief mindfulness-based stress awareness exercises before activity, prompts during exercise that help adolescents link managing physical effort to managing psychological stress, and short post-exercise reflections on stress reduction and mastery experiences. Psychoeducational units on stress physiology and the potential benefits of exercise for coping with stress may further support these aims.

Third, the sequential association involving emotional sensitivity suggests that some adolescents may benefit from activities that help them recognize and manage emotional contagion within group-based exercise. Practical strategies might include exercises that foster awareness of how peers’ emotions influence one’s own feelings, training in interpersonal effectiveness during team activities, and discussions on maintaining appropriate emotional boundaries while preserving empathy. Such components should be implemented with care to avoid pathologizing sensitivity and to respect individual differences in temperament.

#### Implementation within educational settings

For schools, embedding these components into existing structures may enhance feasibility. One option is to integrate stress- and emotion-focused elements within the physical education curriculum, rather than creating entirely new programs. Physical education classes could include brief, structured segments devoted to stress-awareness and emotional regulation skills, with explicit links made between physical and emotional challenges. Teacher training would need to address both basic exercise prescription principles and foundational skills for supporting students’ emotional well-being.

School-wide scheduling is another practical consideration. Short morning exercise sessions, for example, may be helpful in setting a more regulated tone for the school day [102], but academic timetables often leave limited discretionary time. A combined approach—such as short morning routines supplemented by longer sessions later in the day or week—may strike a better balance between academic and health priorities. In addition, systematic assessment and monitoring could be built into multi-week programs, with baseline and follow-up assessments of exercise habits, stress perception, emotional sensitivity, and emotional outcomes, along with interim checks to guide adjustments.

It is important to emphasize that these suggestions are grounded in cross-sectional associations and should be viewed as hypotheses for program development rather than prescriptions. Rigorous evaluation through longitudinal and experimental designs is needed before strong practice recommendations can be made.

### Limitations and methodological considerations

#### Cross-sectional design constraints

The cross-sectional design is the primary methodological limitation of the present study. Because all variables were assessed at a single time point, temporal ordering cannot be established, and the direction of the associations remains uncertain. The patterns observed may reflect exercise predicting later emotional states, emotional states predicting later exercise participation, bidirectional relationships, or shared determinants that influence both. Although alternative models were tested and showed somewhat weaker fit than the hypothesized configuration, such tests cannot substitute for designs that track changes over time [103].

Furthermore, single-time-point assessments are not well suited to capturing day-to-day fluctuations in exercise, stress, and emotions. Ecological momentary assessment or intensive longitudinal designs could examine within-person processes and temporal dynamics that are likely to be obscured in between-person cross-sectional analyses [104]. In view of these limitations, the present results should be interpreted as describing patterns of association rather than causal pathways.

#### Measurement limitations

All key constructs were measured via adolescent self-report, which introduces potential biases related to social desirability, recall inaccuracies, and common method variance. Although diagnostic tests indicated limited common method bias in the current data, multi-method assessment strategies would strengthen confidence in future findings. For example, accelerometer-based measures of physical activity, physiological indices such as cortisol for stress, and behavioral tasks or informant reports for emotional sensitivity could complement subjective evaluations [105].

Emotional sensitivity was operationalized through emotional contagion, capturing the interpersonal aspect of sensitivity. This operationalization was theoretically motivated but necessarily partial. Broader conceptualizations of emotional sensitivity also include intrapersonal sensitivity, sensory processing sensitivity, and the capacity to differentiate emotions. Future studies that incorporate a more comprehensive assessment of these facets may yield a more detailed understanding of how emotional sensitivity co-varies with exercise, stress perception, and emotional outcomes [50].

#### Generalizability boundaries

The sample was drawn from ten schools within a single province in China, which limits the generalizability of the results. Although schools were chosen to reflect variation in urban–rural location and socioeconomic context, the sample may not capture adolescents living in extreme poverty, highly elite educational environments, or other distinctive settings. Adolescents with diagnosed psychiatric conditions and elite athletes were not included, so the findings may not extend to clinical or highly specialized populations.

In addition, the age range of the sample (11–16 years) corresponds primarily to early and middle adolescence. Developmental differences in stress regulation, emotional competencies, and exercise participation patterns suggest that the associations observed here may not generalize to younger children or older adolescents [106]. The model also did not include contextual variables such as family support, peer relationships, or teacher–student relationship quality, which may be associated with both stress perception and emotional sensitivity. Incorporating these factors into future longitudinal and experimental studies would help clarify boundary conditions and provide a stronger basis for examining potential causal processes.

### Future research directions

#### Longitudinal and experimental designs

Future work should prioritize designs that allow temporal sequencing and bidirectional processes to be examined more directly. Prospective longitudinal studies following adolescents over multiple years could use growth curve modelling to test whether changes in exercise are associated with subsequent changes in stress perception and emotional trajectories, while accounting for baseline differences and time-varying confounders [107]. Randomized controlled trials that manipulate exercise parameters and assess the proposed mediators at multiple time points would provide a stronger basis for evaluating potential causal relationships.

Cross-lagged panel models could further clarify whether exercise–emotion associations differ across developmental periods and whether reciprocal pathways strengthen or weaken as adolescents mature [108]. Such designs would also make it possible to examine whether stress perception and emotional sensitivity function as mediators, moderators, or both across time.

#### Mechanistic refinements and extensions

The current model focused on stress perception and emotional sensitivity, but other mechanisms are also plausible. Sleep quality, for example, is linked to both physical activity and emotional regulation and may represent an additional pathway through which exercise is associated with emotional outcomes [109]. Recent studies suggest that improvements in sleep may partially account for links between exercise and reduced emotional difficulties, and that sleep disturbances may mediate the association between stress and emotional symptoms [110].

There is also emerging evidence that combining physical activity with mindfulness-based approaches may be advantageous. For instance, interventions that integrate mindfulness practices with exercise have been associated with larger reductions in emotion dysregulation than either component alone [111]. Other studies indicate that some of the associations between physical activity and emotional outcomes may be statistically explained by sleep quality improvements [112]. Future research could test multi-component programs that jointly target exercise, stress management, emotional skills, and sleep, and examine how these elements interact.

Incorporating neurobiological measures—such as functional neuroimaging, autonomic indices, and neuroendocrine markers—would also help clarify how the psychological pathways identified here relate to underlying biological processes [113]. Integrating psychological and biological data could support more comprehensive biopsychosocial models of how physical exercise is embedded within adolescents’ broader stress and emotion regulation systems.

#### Cultural and contextual variations

Studies conducted in different cultural and educational settings are needed to evaluate the extent to which the association patterns observed here are context-specific. Comparative research across individualistic and collectivistic cultures, and across school systems with varying emphases on competition, examination pressure, and group activities, could help distinguish universal from context-dependent mechanisms [114].

It will also be important to examine potential moderators such as gender, pubertal status, socioeconomic position, and exposure to adverse experiences. These factors may identify subgroups of adolescents for whom exercise is more strongly related to stress perception and emotional outcomes, or for whom additional supports are required. Person-centered approaches, including latent profile analysis, could be used to identify distinct subtypes characterized by different combinations of exercise habits, stress perception, emotional sensitivity, and emotional symptoms [115].

### Conclusion

This study examined how physical exercise, stress perception, and emotional sensitivity are related to negative emotions in a large sample of Chinese adolescents. The pattern of associations was consistent with a model in which higher levels of physical exercise were linked to lower negative emotions both directly and indirectly through lower stress perception, with a smaller sequential pathway involving emotional sensitivity. Stress perception accounted for the largest proportion of the indirect association, and emotional sensitivity appeared to function primarily as a downstream correlate of stress appraisal rather than as a proximal correlate of exercise.

These findings add to the literature by quantifying the relative contributions of direct and indirect pathways and by highlighting the central role of perceived stress in the exercise–emotion association pattern. They also suggest that emotional sensitivity, as operationalized through emotional contagion, may represent an additional interpersonal process to consider when examining adolescent emotional health. While the cross-sectional design does not allow causal inferences, the results indicate that physical exercise, stress perception, and emotional sensitivity co-occur in ways that may be informative for theory and for the design of future interventions.

From a practical perspective, the results point to physical exercise as a potentially important behavioral resource (i.e., a modifiable lifestyle behavior) to consider in efforts to support adolescent emotional well-being, particularly when programs explicitly attend to stress management and interpersonal emotional processes. Future longitudinal and experimental studies conducted in diverse cultural and educational settings are needed to test the temporal ordering and causal status of these pathways and to determine how best to incorporate exercise into comprehensive strategies for promoting adolescent mental health.

## Data Availability

The datasets generated and/or analysed during the current study are available from the corresponding author on reasonable request.

## Abbreviations

AIC: Akaike information criterion
AVE: Average variance extracted
BDNF: Brain-derived neurotrophic factor
BIC: Bayesian information criterion
CFA: Confirmatory factor analysis
CFI: Comparative fit index
CI: Confidence interval
CR: Composite reliability
ES: Emotional sensitivity
ESIM: Emotional Sensitivity Integration Model
HPA: Hypothalamic–pituitary–adrenal
HTMT: Heterotrait–monotrait ratio of correlations
MI: Modification index
NE: Negative emotions
PANAS: Positive and Negative Affect Schedule
PE: Physical exercise
RMSEA: Root mean square error of approximation
SD: Standard deviation
SE: Standard error
SEM: Structural equation modeling
SP: Stress perception
SPSS: Statistical Package for the Social Sciences
SRMR: Standardized root mean square residual
TLI: Tucker–Lewis index
